# Eating Speed and Incidence of Diabetes in a Japanese General Population: ISSA-CKD

**DOI:** 10.3390/jcm10091949

**Published:** 2021-05-01

**Authors:** Hideyuki Fujii, Shunsuke Funakoshi, Toshiki Maeda, Atsushi Satoh, Miki Kawazoe, Shintaro Ishida, Chikara Yoshimura, Soichiro Yokota, Kazuhiro Tada, Koji Takahashi, Kenji Ito, Tetsuhiko Yasuno, Shota Okutsu, Shigeaki Mukoubara, Hitoshi Nakashima, Shigeki Nabeshima, Seiji Kondo, Masaki Fujita, Kosuke Masutani, Hisatomi Arima, Daiji Kawanami

**Affiliations:** 1 Department of Endocrinology and Diabetes Mellitus, Fukuoka University School of Medicine, Fukuoka 814-0180, Japan; joeten1cho@yahoo.co.jp; 2 Department of Preventive Medicine and Public Health, Faculty of Medicine, Fukuoka University, Fukuoka 814-0180, Japan; shunsuke.funakoshi@gmail.com (S.F.); tmaeda@fukuoka-u.ac.jp (T.M.); atsushis@fukuoka-u.ac.jp (A.S.); miki1024@fukuoka-u.ac.jp (M.K.); ychikara@fukuoka-u.ac.jp (C.Y.); harima@fukuoka-u.ac.jp (H.A.); 3 Department of Oral and Maxillofacial Surgery, Faculty of Medicine, Fukuoka University, Fukuoka 814-0180, Japan; shintarou19910118@gmail.com (S.I.); kondo@fukuoka-u.ac.jp (S.K.); 4 Division of Nephrology and Rheumatology, Department of Internal Medicine, Faculty of Medicine, Fukuoka University, Fukuoka 814-0180, Japan; syokota@fukuoka-u.ac.jp (S.Y.); ktada@fukuoka-u.ac.jp (K.T.); reply_09030728174@yahoo.co.jp (K.T.); kito@fukuoka-u.ac.jp (K.I.); yasuno9584@fukuoka-u.ac.jp (T.Y.); hitscenenakashima@gmail.com (H.N.); kmasutani@fukuoka-u.ac.jp (K.M.); 5 Department of General Medicine, Fukuoka University Hospital, Fukuoka 814-0180, Japan; shota.o.19870528@gmail.com (S.O.); snabeshi@fukuoka-u.ac.jp (S.N.); 6 Department of Internal Medicine, Nagasaki Prefecture Iki Hospital, Nagasaki 811-5132, Japan; s-mukoubara@ikihp.jp; 7 Department of Respiratory Medicine, Faculty of Medicine, Fukuoka University, Fukuoka 814-0180, Japan; mfujita@fukuoka-u.ac.jp

**Keywords:** diabetes, eating speed, primary prevention, lifestyle

## Abstract

Background: We investigated whether eating speed was associated with the incidence of diabetes in a Japanese general population. Methods: A total of 4853 Japanese individuals without diabetes at baseline were analyzed. Self-reported eating speed was categorized as slow, medium, and fast on the basis of questionnaire responses. The study outcome was the incidence of diabetes. Results: After an average follow-up period of 5.1 years, 234 individuals developed diabetes. The incidence of diabetes per 1000 person-years was 4.9 in the slow eating speed group, 8.8 in the medium eating speed group, and 12.5 in the fast eating speed group, respectively (*** *p* < *0*.001 for trend). The HRs were 1.69 (95%CI 0.94–3.06) for the medium eating speed and 2.08 (95%CI 1.13–3.84) for the fast eating speed, compared to the slow eating speed (* *p* = *0*.014 for trend) after adjustment for age, gender, smoking status, drinking, exercise, obesity, hypertension, and dyslipidemia. Conclusion: Faster eating speed increased a risk for the incidence of diabetes in a general Japanese population.

## 1. Introduction

Diabetes is a life-threatening disease that causes microvascular and macrovascular complications [1,2,3,4,5,6]. Diabetes is considered as a serious disorder that doubles the risk of premature death [7]. A longitudinal study demonstrated that the incidence rate of coronary artery disease per 1000 person-years in Japanese patients with type 2 diabetes was 9.59, which is approximately three times higher than the general population [8]. In Japan, diabetic kidney disease is the leading cause (43.5%) among new dialysis patients [9]. The number of people with diabetes and impaired glucose tolerance in Japan is estimated at 20 million, and this number has been increasing since 1997 [10]. According to the 2016 National Health and Nutrition Survey by the Japanese Ministry of Health, Labor and Welfare, the prevalence rate of type 2 diabetes in Japan was 12.1%. The effective prevention of type 2 diabetes requires up-to-date knowledge of risk factors for the disease.

It has been shown that interventions seeking to impact lifestyle behaviors, including improving dietary and exercise habits, can prevent the onset of type 2 diabetes [11,12,13]. Obesity [11,12,13,14] and insufficient exercise have been implicated as established modifiable risk factors for type 2 diabetes [15,16,17], as well as impaired glucose tolerance, smoking [18], alcohol intake [19,20], and inadequate diet (calorie intake and content) [11,12,13,21,22,23]. Several studies have shown that fast eating is associated with the increased risk of type 2 diabetes [24,25,26]. Previous studies have used questionnaires to classify eating speed. For example, the question was “Do you eat faster than people who eat together at the same table?” or “Do you eat faster than people of the same generation?” In one study, the eating speed was classified into five groups (very slow, relatively slow, medium, relatively fast, and very fast) [25]. However, the evidence on this topic is mainly derived from case–control studies or studies conducted among special populations (e.g., worksite populations), and it is unclear to what extent this evidence is generalizable to general populations. The aim of this large-scale population-based study was to examine the effect of eating speed on the development of diabetes in a general population in Japan.

## 2. Materials and Methods

### 2.1. Study Design

The Iki City Epidemiological Study of Atherosclerosis and Chronic Kidney Disease (ISSA-CKD) is a population-based retrospective cohort study that uses annual health checkup data for the citizens of Iki City, Nagasaki Prefecture, Japan. ISSA-CKD has been described in the accompanying literatures [27,28,29,30]. The present study was conducted according to the guidelines of the Declaration of Helsinki of 1975, revised in 2013, and approved by the Fukuoka University Clinical Research and Ethics Center (No.2017M010).

### 2.2. Participants

A total of 7895 individuals received annual health checkups from 2008–2017. Of these people, 3042 (38.5%) were excluded: 1881 dropped out from consecutive follow-up annual medical checkups, and 1161 had diabetes at baseline. Thus, 4853 citizens were analyzed in this study.

### 2.3. Data Collection

At baseline, we collected information on eating speed, using a questionnaire with the following question: “How fast is your eating speed compared with others?” The response categories were slow, medium, and fast. Information on smoking, alcohol drinking, regular exercise, family history of diabetes, and current use of medications for hypertension, dyslipidemia, and diabetes was also collected via questionnaire. We defined obesity as a BMI ≥25 kg/m^2^. Participants who had smoked 100 cigarettes or more, or who had smoked regularly for 6 months and more were defined as currently smoking. Drinking behavior was defined as drinking on 5 days or more per week. Regular exercise was defined as exercising ≥30 min/day at least twice a week. Hypertension was defined as a systolic blood pressure of 140/90 mmHg or more or use of blood pressure-lowering medicine. Fasting or casual blood and urine samples were collected. Plasma glucose levels were measured by an enzymatic method, and glycated hemoglobin (HbA1c) levels (National Glycohemoglobin Standardization Program value) were determined by a high-performance liquid chromatography method. The diagnosis of diabetes was determined by a fasting glucose level ≥ 6.99 mmol/L, casual blood glucose level ≥ 11.10 mmol/L, HbA1c ≥ 6.5%, or the use of glucose-lowering therapies. Serum low-density lipoprotein (LDL) cholesterol, high-density lipoprotein (HDL) cholesterol and triglyceride concentrations were measured enzymatically. Dyslipidemia was defined as LDL cholesterol ≥ 3.62 mmol/L, HDL cholesterol < 1.03 mmol/L, triglycerides ≥ 1.69 mmol/L or the use of lipid-lowering medication.

### 2.4. Outcome

The incidence of diabetes (fasting glucose level ≥ 6.99 mmol/L, casual blood glucose level ≥ 11.10 mmol/L, HbA1c ≥ 6.5%, or the use of glucose-lowering therapies) at the end of follow-up.

### 2.5. Statistical Analysis

Continuous variables were expressed as means ± SD. Simple regression models were used to determine trends across tertile groups of eating speed. Categorical variables were expressed as the number (percentage) of participants. Logistic regression models were used to test trends across groups. Incidence rates of diabetes were expressed by person-year. We estimated crude and multivariable-adjusted hazard ratios (HRs) and their 95% confidence intervals (CIs) of the effect of eating speed on the development of diabetes by the use of Cox proportional hazards models. Then, we next adjusted for age, sex, smoking status, alcohol drinking, exercise, obesity, hypertension and dyslipidemia. A two-tailed *p* value of less than 0.05 was considered statistically significant. Analyses were performed using SAS, Version 9.4.

## 3. Results

The average age of the participants at baseline was 59.6 years, 55.5% were women, and the average BMI was 23.6 kg/m^2^. The mean baseline fasting blood glucose level was 5.1 ± 0.5 mmol/L, and the mean HbA1c level was 5.1 ± 0.4%. A total of 1350 people (27.8%) were classified in the fast eating speed group, 2993 (61.7%) were classified in the medium eating speed group, and 510 (10.5%) were classified in the slow eating speed group. Table 1 shows the baseline characteristics. A self-reported faster eating speed was associated with younger age, higher BMI, higher triglycerides, and lower levels of HDL-cholesterol.

During an average follow-up of 5.1 years (24,745 person-years), 234 individuals developed diabetes (incidence rate: 9.4 per 1000 person-years). Table 2 shows the risks of diabetes by reported eating speed. The incidence rates (per 1000 person-years) were 4.9 for the slow eating speed group, 8.8 for the medium eating speed group, and 12.5 for the fast eating speed group (*p* < 0.001 for trend). These associations remained statistically significant even after adjustment for age, gender, smoking status, drinking habits, exercise habits, obesity, hypertension, and dyslipidemia: The multivariable-adjusted HRs (95% CIs) were 1.69 (0.94–3.06) for medium eating speed, and 2.08 (1.13–3.84) for fast eating speed, compared with the reference group of slow eating speed (* *p* = 0.014 for trend). When BMI (instead of obesity), systolic blood pressure (instead of hypertension), HDL-c and triglycerides (instead of dyslipidemia) were included in multivariable analysis as covariates, the hazard ratios were 1.72 (95% CIs 0.95–3.11) for medium eating speed and 1.94 (95% CIs 1.05–3.58) for fast eating speed compared with slow eating speed. When waist circumference (instead of BMI) was included in multivariable analysis as covariate, the hazard ratios were 1.72 (95% CIs 0.94–3.08) for medium eating speed and 2.05 (95% CIs 1.15–3.78) for fast eating speed compared with slow eating speed.

Table 3 shows the results of the subgroup analysis. The effect of reported eating speed on the development of diabetes was comparable across the subgroups defined by age, gender, obesity, hypertension, dyslipidemia, smoking, drinking habits, and regular exercise (all *p* > 0.1 for the interactions).

## 4. Discussion

In this large-scale observational study of a general Japanese population, a self-reported faster eating speed was associated with a higher risk of developing diabetes. This association remained significant in the multivariable analysis, including age, sex, smoking status, drinking, regular exercise, obesity, hypertension and dyslipidemia as covariates. The correlation of eating speed with incidence of diabetes was comparable across subgroups defined by age, sex, obesity, hypertension, dyslipidemia, current smoking and drinking.

Previous evidence on the relationship between eating speed and the risk of type 2 diabetes is mainly derived from case–control studies. A case–control study conducted in Lithuania compared 234 individuals with newly diagnosed type 2 diabetes with 468 controls, demonstrating that the risk of type 2 diabetes was more than doubled for people who ate quickly compared with others [31]. In Japan, Sakurai et al. [25] reported that eating quickly increased the risk of diabetes among 2050 middle-aged Japanese male workers undergoing medical examinations. In a 3-year longitudinal study of 172 people in Japan who underwent medical examinations in a single hospital, Totsuka et al. [32] found that self-reported fast eating speed was associated with the incidence of impaired glucose tolerance, which was confirmed using a 75 g glucose tolerance test. One large-scale population-based study of a Japanese population who underwent annual health checkups reported a 1.12-fold higher risk of diabetes in the group of fast eating speed than in the combined group of medium and slow eating speed during 1-year to 3-year follow-up [26]. The present large-scale population-based longitudinal study with long-term follow-up (average 5.1 years) confirmed the findings of previous studies and clearly demonstrated a strong, linear relationship between self-reported eating quickly and the development of diabetes (multivariable-adjusted HRs 1.69 for medium eating speed and 2.08 for fast eating speed compared with the reference group of slow eating speed, * *p* = 0.014 for trend) among a general Japanese population.

The precise mechanisms by which eating speed increases the incidence of diabetes have not been clearly defined, but one possible explanation for the effect is the development of insulin resistance through weight gain. Fast eating has been shown to lead to weight gain, obesity [25,32,33,34,35,36,37,38,39], and the subsequent development of insulin resistance [24,25,32,39]. Second, fast eating may cause postprandial hyperglycemia. It has been reported that, in healthy subjects, thorough mastication was associated with lower levels of postprandial blood glucose compared with normal mastication [40]. Therefore, fast eating, which is associated with lower mastication, may cause postprandial hyperglycemia. Over time, postprandial hyperglycemia may gradually cause pancreatic β-cell exhaustion, leading to a decrease in insulin secretion [41]. Third, a decrease in mastication may lead to an increase in food intake. An animal study found that, in rats, thorough mastication activated histamine in the hypothalamus and binding of histamine to H1 receptors in the paraventricular nucleus and ventromedial lobe of the hypothalamus resulted in food intake suppression [42]. Thus, fast eating, which is associated with decreased mastication, may increase food consumption. Fourth, decreases in the secretion of peptide YY and glucagon-like peptide 1 (GLP-1) by fast eating may cause postprandial hyperglycemia [43]. Fifth, fast eating may be associated with a delayed feeling of fullness and satiety, which leads to over-eating. A previous study reported that slow eating speed reduced ghrelin secretion in response to carbohydrate load in obese adolescents [44]. Furthermore, Rigamonti et al. reported that slow feeding rates increased peptide YY and GLP-1 secretion [43,45]. Taken together, fast eating may cause these changes in hormone secretion, leading to a delay in the feeling of fullness and satiety, which leads to over-eating.

The strengths of the present study were its relatively large sample size and population-based longitudinal design. In addition, the onset of diabetes was evaluated by blood glucose and HbA1c levels at annual medical examinations. Some previous studies have evaluated the onset of diabetes based only on self-reported information. The present study has several limitations. First, eating speed was self-reported and was not objectively evaluated. The accuracy of evaluating eating speed based on self-report is controversial. Woodland et al. demonstrated that the match rate of self-reported eating speed and the objective measure of eating rate was 47.4% [46]. A future study using a reliable method to assess eating speed will be required to obtain more objectivity. Second, a detailed nutritional survey was not conducted in this study. Third, people who are interested in their own health are more likely to undergo medical examinations than those who are not. Our findings obtained from participants of the ISSA-CKD study do not always apply to the general population. Further study will be interesting to elucidate whether or not similar results can be observed in the general Japanese population. Fourth, no information was available on the etiological type of diabetes, although most onsets after age 40 are type 2 diabetes [47,48]. Fifth, a detailed amount of exercise was not available. However, previous studies have shown that exercise (≥4 METs/h/week) of at least 30 min per week on at least 2 days a week is the minimum required to improve physical fitness and musculo-skeletal function [49]. We created the questionnaire about exercise habits on this basis. A future study using a reliable method to assess exercise habits and physical fitness index will be required.

## 5. Conclusions

In conclusion, a self-reported faster eating speed was clearly associated with a higher risk of developing diabetes in this large-scale observational study of a general Japanese population. A feasible strategy in the future is to work with physicians and registered dietitians to provide nutrition therapy to improve eating speed during medical examination. The population strategy to reduce eating speed appears to provide further protection against the emerging burden of diabetes.

## Figures and Tables

**Table 1:** Baseline characteristics by self-reported eating speed.

	Self-Reported Eating Speed			
	Slow	Medium	Fast	*p* Value for Trend
	(*N* = 510)	(*N* = 2993)	(*N* = 1350)	
Age, mean (SD), years	61.6 (±10.7)	59.8 (±10.5)	58.5 (±10.8)	*** < 0.001
Male, N/total N (%)	180/510 (35.3%)	1271/2993 (42.5%)	709/1350 (52.5%)	*** < 0.001
Smoking status, N/total N (%)				
Never smoker	423/510 (82.9%)	2275/2993 (76.0%)	975/1350 (72.2%)	*** < 0.001
Ex-smoker	19/510 (3.7%)	151/2993 (5.0%)	89/1350 (6.6%)	
Current smoker, <20 cigarettes/day	17/510 (3.3%)	134/2993 (4.5%)	72/1350 (5.3%)	
Current smoker, ≥20 cigarettes/day	22/510 (4.3%)	226/2993 (7.6%)	129/1350 (9.6%)	
Current smoker, missing information on the number of cigarettes/day	29/510 (5.7%)	207/2993 (6.9%)	85/1350 (6.3%)	
Alcohol intake ^†^, N/total N (%)				
No	305/505 (60.4%)	1609/2970 (54.2%)	649/1342 (48.4%)	** 0.004
Occasional alcohol drinking	100/505 (19.8%)	680/2970 (22.9%)	347/1342 (25.9%)	
Daily current alcohol drinking, <20 g/day	43/505 (8.5%)	221/2970 (7.4%)	97/1342 (7.2%)	
Daily current alcohol drinking, 20–39.9 g/day	39/505 (7.7%)	318/2970 (10.7%)	182/1342 (13.6%)	
Daily current alcohol drinking, ≥40 g/day	18/505 (3.6%)	142/2970 (4.8%)	67/1342 (5.0%)	
Regular exercise ^‡^, N/total N (%)	120/510 (23.5%)	809/2993 (27.0%)	357/1350 (26.4%)	0.451
Body mass index, mean (SD), kg/m^2^	22.7 (±3.3)	23.3 (±3.3)	24.5 (±3.6)	*** < 0.001
Obesity ^§^, N/total N (%)	101/510 (19.8%)	815/2993 (27.2%)	554/1350 (41.0%)	*** < 0.001
Systolic blood pressure, mean (SD), mmHg	128.7 (±19.6)	129.0 (±18.3)	128.9 (±19.0)	0.987
Diastolic blood pressure, mean (SD), mmHg	73.8 (±10.8)	74.8 (±11.1)	75.6 (±11.3)	** 0.002
High-density lipoprotein cholesterol, mean (SD), mmol/L	1.63 (±0.41)	1.62 (±0.42)	1.55 (±0.41)	*** < 0.001
Low density lipoprotein cholesterol, mean (SD), mmol/L	3.10 (±0.82)	3.18 (±0.81)	3.20 (±0.82)	0.06
Triglyceride, mean (SD), mmol/L	1.29 (±0.93)	1.28 (±0.84)	1.44 (±1.03)	*** < 0.001
Dyslipidemia ^¶^, N/total N (%)	194/510 (38.0%)	1244/2993 (41.6%)	642/1350 (47.6%)	*** < 0.001
Hypertension ^††^, N/total N (%)	209/510 (41.0%)	1272/2993 (42.5%)	588/1350 (43.6%)	0.308
HbA1c, mean (SD),%	5.1 (±0.3)	5.1 (±0.4)	5.1 (±0.4)	0.669
Fasting blood glucose (SD), mmol/L ^†††^	5.0 (±0.5)	5.0 (±0.5)	5.1 (±0.6)	** 0.0013

^†^ Habitually drinking on 5 or more days per week. ^‡^ Habitually exercising ≥ 30 min per day twice or more per week. ^§^ Body mass index ≥ 25 kg/m^2^. ^¶^ Low-density lipoprotein cholesterol ≥ 3.62 mmol/L, high-density lipoprotein cholesterol < 1.03 mmol/L, triglycerides ≥ 1.69 mmol/L, or use of lipid-lowering medication. ^††^ Systolic blood pressure ≥ 140 mmHg, diastolic blood pressure ≥ 90 mmHg, or use of blood pressure-lowering medication. ^†††^ Available for 381 participants in the slow group, 2230 in the medium group, and 1017 in the fast group. ** *p* < 0.05, *** *p* < 0.001.

**Table 2:** Risk of diabetes mellitus by self-reported eating speed.

	Self-Reported Eating Speed			
	Slow	Medium	Fast	*p* Value for Trend
	(N = 510)	(N = 2993)	(N = 1350)	
N of events/person-years	12/2468	134/15,234	88/7034	
Incidence rate (per 1000 person-years)	4.9	8.8	12.5	
Crude hazard ratio	1	1.82	2.61	*** < 0.001
(95% Confidence interval)	(Reference)	(1.01–3.29)	(1.43–4.77)	
Adjusted hazard ratio ^†^	1	1.69	2.08	** 0.014
(95% Confidence interval)	(Reference)	(0.94–3.06)	(1.13–3.84)	

^†^ Adjusted for age, sex, smoking status, alcohol drinking, exercise, obesity, hypertension and dyslipidemia. ** *p* < 0.05, *** *p* < 0.001.

**Table 3:** Subgroup analysis.

	Self-Reported Eating Speed			
	Slow	Medium	Fast	*p* Value for Interaction
	(*N* = 510)	(*N* = 2993)	(*N* = 1350)	
Age				
<65 years	1 (reference)	1.04 (0.48–2.29)	1.52 (0.68–3.36)	0.105
≥65 years	1 (reference)	2.64 (1.06–6.55)	2.61 (1.01–6.79)	
Sex				
Male	1 (reference)	2.48 (0.91–6.80)	3.03 (1.09–8.42)	0.617
Female	1 (reference)	1.28 (0.61–2.68)	1.57 (0.72–3.43)	
Obesity				
Yes	1 (reference)	1.35 (0.54–3.37)	1.94 (0.77–4.87)	0.462
No	1 (reference)	1.97 (0.91–4.29)	2.02 (0.88–4.61)	
Hypertension				
Yes	1 (reference)	1.91 (0.83–4.40)	2.31 (0.98–5.45)	0.895
No	1 (reference)	1.47 (0.63–3.41)	1.74 (0.73–4.17)	
Dyslipidemia				
Yes	1 (reference)	1.77 (0.71–4.41)	2.39 (0.95–6.02)	0.402
No	1 (reference)	1.76 (0.80–3.84)	1.88 (0.82–4.31)	
Current smoking				
Yes	1 (reference)	1.26 (0.38–4.12)	1.10 (0.31–3.84)	0.349
No	1 (reference)	1.82 (0.92–3.61)	2.48 (1.23–5.00)	
Daily alcohol intake				
Yes	1 (reference)	5.20 (0.71–37.88)	5.33 (0.71–39.81)	0.298
No	1 (reference)	1.33 (0.71–2.49)	1.81 (0.94–3.46)	
Regular exercise				
Yes	1 (reference)	1.63 (0.50–5.31)	2.63 (0.79–8.70)	0.662
No	1 (reference)	1.63 (0.82–3.25)	1.81 (0.89–3.69)	

Values are hazard ratios (95% confidence intervals) adjusted for age (except for the subgroup analysis by age), sex (except for the subgroup analysis by sex),obesity (except for the subgroup analysis by obesity), hypertension (except for the subgroup analysis by hypertension), dyslipidemia (except for the subgroup analysis by dyslipidemia), current smoking (except for the subgroup analysis by current smoking), daily alcohol drinking (except for the subgroup analysis by alcohol drinking) and regular exercise (except for the subgroup analysis by regular exercise). Obesity: body mass index ≥ 25 kg/m^2^. Hypertension: systolic blood pressure ≥ 140 mmHg, diastolic blood pressure ≥ 90 mmHg or use of blood pressure-lowering medication. Dyslipidemia: low-density lipoprotein cholesterol ≥ 3.62 mmol/L, high-density lipoprotein cholesterol < 1.03 mmol/L, triglycerides ≥ 1.69 mmol/L, or the use of lipid-lowering medication.

## Data Availability

The data presented in this study are available on request from the corresponding author. The data are not publicly available in order to preserve the anonymity of the subjects involved in the study.
